# Supplementary material for: Evidence for loss of contractile phenotype of the mouse aortic vascular smooth muscle (MOVAS) cell line with increasing number of passages in vitro
Source: PLoS One. 2025 Dec 19;20(12):e0339118. doi: 10.1371/journal.pone.0339118 (PMC12716690; doi:10.1371/journal.pone.0339118)
Supplement: S1 File — (DOCX) [file pone.0339118.s001.docx]

**Supplemental data**

**Methods**

*Characterization of cell morphology using the ImageJ plugin MorphoLibJ:*

MorphoLibJ plugin was applied to pictures of MOVAS cells at P3, P5 and P8 (example shown in Fig.1A). This plugin allows the measurement of cell area and perimeter, but also lengthening, which corresponds to the ratio of the length of the longest axis to the length of the shortest axis. These parameters are depicted in the table below.

| **Cells** | 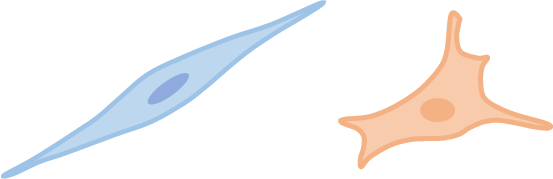 |
| --- | --- |
| **Area**  *(µm²)* | 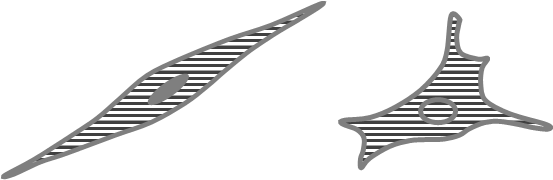 |
| **Perimeter**  *(µm)* | 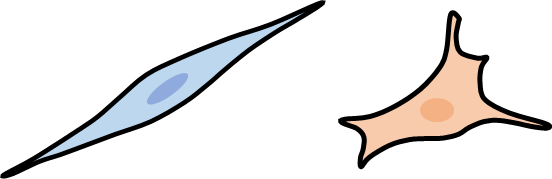 |
| **Lengthening**  *(arbitrary units)*  a  b | 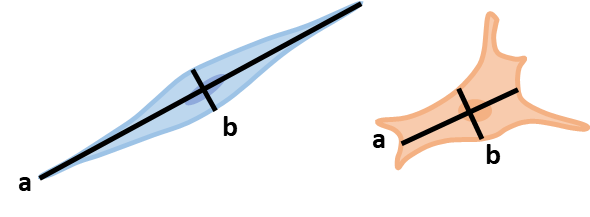  *a : length of the longest axis / b : length of the shortest axis* |

*AFM calibration and settings for measurements of YM values:*

The QNM-LC-CAL probes were chosen since their specs match the requirements of live cells imaging (tip height of 16 µm, tip radius of 65 nm and spring constant of approximately 0.1 N/m). According to Hook’s law, the nominal force applied to the sample can be expressed as followed:

F = k x d

Where k is the spring constant, and d the tip/sample distance. F must be expressed in Newtons so that the Young’s modulus can be further determined. First, an approach/retract curve is recorded on a non-compliant sample (typically a glass slide) in the same medium as the imaging medium, which returns the cantilever’s deflection sensitivity expressed in nm/V. Second, after withdrawing the probe from the surface by a few µm, the cantilever is tuned from zero to 300 kHz to calculate the spring constant expressed in N/m. This second step is recommend although k is given by the manufacturer since a small discrepancy can be observed. The tip radius can be double checked by using a tipcheck sample.

In order to obtain a valid mechanical response, we applied a force ranging from 150 to 500 pN, sufficient to indent into the cell by 100 to 200 nm and small enough to avoid any invasiveness that could amount to stress or even cell death.

YM measurement: We used a Sneddon approximation to extract the Young’s modulus. This geometrical model is often used on biological samples like living cells. The force (in N) applied to the sample can be represented as follows:

F = 2 Es / [(π (1-vs²)] . tan α . δ2

Es is the sample’s Young’s modulus (in Pa)

vs is the sample’s Poisson’s ratio

α is the tip half angle

δ is the indentation depth

If the tip has been correctly calibrated, Es can automatically be extracted from the linear portion of each extension force curve. The obtained Young’s modulus is proportional to the sample’s capacity to deform under a mechanical pressure. By convention, the color code of AFM Young’s modulus images I such that the brighter the contrast, the higher the Young’s modulus. For more details on the force curve processing, we used a baseline correction algorithm to correct the sligh tilt often observed on the force curves, and the “Best Estimate” feature: basically, a line drawn between the first and last points of the force curve is subtracted from each point in the force curve, effectively rotating it. The point with the resulting minimum value is selected as the contact point. This method emphasizes the minimum force at the contact point while de-emphasizing forces due to noise or interference in the non-contact region, reducing the likelihood that the wrong point is selected.
