# Supplementary material for: Evidence for loss of contractile phenotype of the mouse aortic vascular smooth muscle (MOVAS) cell line with increasing number of passages in vitro
Source: PLoS One. 2025 Dec 19;20(12):e0339118. doi: 10.1371/journal.pone.0339118 (PMC12716690; doi:10.1371/journal.pone.0339118)
Supplement: S1 Table — (DOCX) [file pone.0339118.s002.docx]

*Table S1. Sequences of primers used for RT-qPCR analyses:*

| **Genes** | | **Primers** | **Efficiency** | **Product Length (bp)** | **Temperature** |
| --- | --- | --- | --- | --- | --- |
| *Eef1a1* | Elongation factor 1-alpha 1 | F: 5’- ACGAGGCAATGTTGCTGGTGAC - 3’ | 102.3% | 132 | 60°C |
|  |  | R: 5’ – GTGTGACAATCCAGAACAGGAGC - 3’ |  |  |  |
| *Acta2* | Actin, aortic smooth muscle | F: 5’ - GTACCACCATGTACCCAGGC - 3’ | 108.3% | 166 | 60°C |
|  |  | R: 5’ - GCTGGAAGGTAGACAGCGAA - 3’ |  |  |  |
| *Cnn1* | Calponin-1 | F: 5’ - GTTGCGCTTGTCTGTGTCAT - 3’ | 110.5% | 187 | 60°C |
|  |  | R: 5’ - CTCCCGCTGATGGTCGTATT - 3’ |  |  |  |
| *Myocd* | Myocardin | F: 5’ - TCTGCCGATGGATTCTTCCGTG - 3’ | 106.3% | 110 | 62°C |
|  |  | R: 5’ - AGAGCCCATCTCTACTGCTGT C - 3’ |  |  |  |
| *Lgals3* | Galectin-3 | F: 5’ - AACACGAAGCAGGACAATAACTGG - 3’ | 110.5% | 189 | 60°C |
|  |  | R: 5’- GCAGTAGGTGAGCATCGTTGAC - 3’ |  |  |  |
| *Klf4* | Krüppel-like factor 4 | F: 5’ – CTATGCAGGCTGTGGCAAAACC – 3’ | 107.8% | 157 | 60 °C |
|  |  | R: 5’ - TTGCGGTAGTGCCTGGTCAGTT – 3’ |  |  |  |
