# Supplementary material for: Evidence for loss of contractile phenotype of the mouse aortic vascular smooth muscle (MOVAS) cell line with increasing number of passages in vitro
Source: PLoS One. 2025 Dec 19;20(12):e0339118. doi: 10.1371/journal.pone.0339118 (PMC12716690; doi:10.1371/journal.pone.0339118)
Supplement: S1 Fig — RT-qPCR analysis of gene expression of contractile (Acta2, Cnn1, Myocd) vs synthetic (Lgals3, Klf4) phenotype markers in MOVAS cells at different passages were pooled in the same plot. Regression lines were calculated and a Pearson correlation test was used to determine the statistical significance and the r coefficient (indicated within each graph). The regression lines are represented by black lines, with 95% confidence intervals shown in grey. (DOCX) [file pone.0339118.s003.docx]

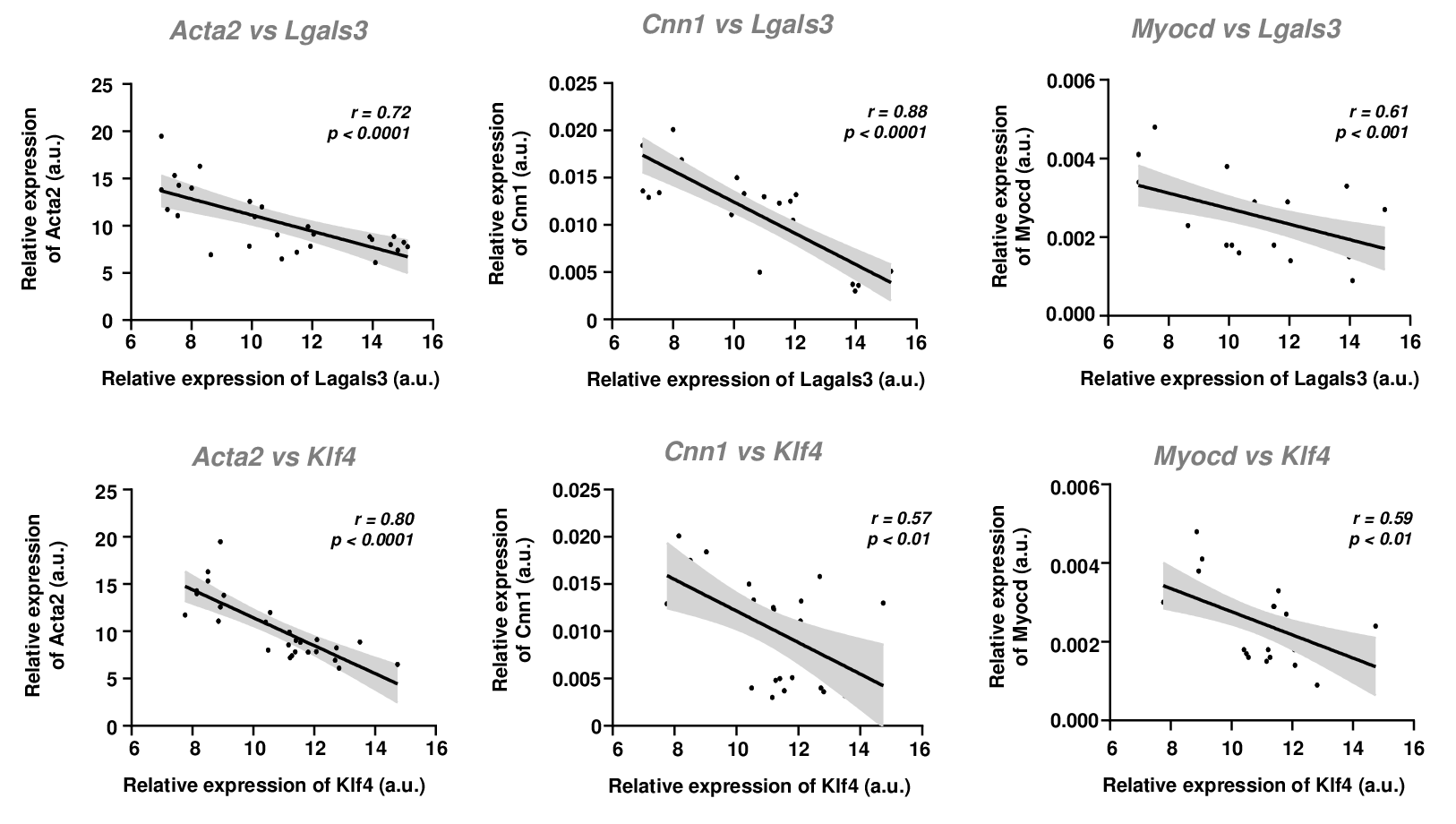


**Fig. S1. Relative expression of contractile *vs* synthetic markers in MOVAS cells.** RT-qPCR analysis of gene expression of contractile (*Acta2*, *Cnn1, Myocd)* *vs* synthetic (*Lgals3, Klf4)* phenotype markers in MOVAS cells at different passages were pooled in the same plot. Regression lines were calculated and a Pearson correlation test was used to determine the statistical significance and the r coefficient (indicated within each graph). The regression lines are represented by black lines, with 95% confidence intervals shown in grey.
