# Supplementary material for: Evidence for loss of contractile phenotype of the mouse aortic vascular smooth muscle (MOVAS) cell line with increasing number of passages in vitro
Source: PLoS One. 2025 Dec 19;20(12):e0339118. doi: 10.1371/journal.pone.0339118 (PMC12716690; doi:10.1371/journal.pone.0339118)
Supplement: Attachment — Submitted filename: Response to reviewers-PONE-D-25-33029 v2.docx [file pone.0339118.s006.docx]

**PONE-D-25-33029**

Cadoret et al. - Evidence for loss of contractile phenotype of the mouse aortic vascular smooth muscle (MOVAS) cell line with increasing number of passages in vitro.

Responses to reviewers:

*We thank the three reviewers for their constructive comments which helped us to improve this manuscript. The changes made to the manuscript have been highlighted in yellow.*

**Reviewer #1:**

The manuscript titled "Evidence for Loss of Contractile Phenotype of the Mouse Aortic Vascular Smooth Muscle (MOVAS) Cell Line with Increasing Number of Passages In Vitro" is well-structured and logically presented. It addresses a critical aspect of vascular smooth muscle cell (VSMC) research, emphasizing the importance of monitoring cell phenotype in vitro. The data is robust, and the conclusions are well-supported.

*We thank Reviewer #1 for this nice comment.*

To improve the manuscript, ensure consistent use of abbreviations (e.g., VSMCs, MOVAS) and correct minor grammatical and typographical errors.

*Answer: We carefully reviewed the manuscript and ensured that the correct abbreviations were used to avoid any confusion. We also indicated in several places (particularly in the Mat&Meth section) that the term "MOVAS cells" was used. Finally, the revised manuscript was reviewed by several people to correct any typos or grammatical errors.*

Including a brief mention of future research directions in the conclusion would provide a broader perspective.

*Answer: Conclusion has been reworded in order to provide a broader perspective and recommendations for researchers were provided at the end of the conclusion especially by inviting them to perform a regular characterization of cell phenotype.*

Add background information on the common use and advantages of MOVAS cells over primary VSMCs and explain the significance of maintaining the contractile phenotype for cardiovascular research.

*Answer: This information has been added in the manuscript at several places: page 4 (second paragraph) and page 13 (first paragraph).*

Clearly state the hypothesis or research question and describe the statistical methods used, including specific tests and significance criteria.

*Answer: The main hypothesis has been mentioned at the end of the introduction section. The paragraph dealing with statistical methods has been completed by adding missing information.*

Expand the discussion to compare findings with other studies and place them in the broader context of VSMC research.

*Answer: The sections of the discussion comparing the results with those of other studies and placing them within the broader context of VSMC research have been highlighted in yellow. Some references have also been added to update the discussion.*

Discuss potential study limitations and suggest how they could be addressed in future research. Highlight specific applications or implications for cardiovascular disease research or treatment.

*Answer: A paragraph describing the major limitation of the study and specifically addressing the problem of differences between vial batches has been added at the end of the discussion section of the revised manuscript.*

Provide recommendations for researchers using MOVAS cells, ensure all references are current and relevant, and consider adding more references to support key points.

*Answer: Conclusion has been reworded and recommendations for researchers have been added. More recent references have been added in the introduction and discussion sections.*

Include details on Atomic Force Microscopy (AFM) calibration and settings for Young's modulus mapping.

*Answer: Details about AFM calibration and YM mapping settings have been added to the supplemental data file.*

Describe statistical tests in the methods section, detailing the number of replicates and sample sizes. Clarify the rationale for choosing non-parametric ANOVA and Mann-Whitney's U test, ensuring their appropriateness given the data distribution.

*Answer: Before comparing the datasets, the distribution of values was systematically tested using the D'Agostino & Pearson's normality test, which showed that the distribution was normal in some cases but not in others. For this reason, we chose to use non-parametric tests in all cases for the sake of consistency and also because the number of replicates was not always sufficient to safely apply parametric tests. A sentence mentioning the verification of the distribution of values and the choice of non-parametric tests was added to the Mat&Meth section. In addition, the number of replicates was systematically added to the figure legends.*

**Reviewer #2:**

I believe the manuscript is a very straight forward study that clearly shows evidence for the phenotypic shift of the Movas cells with advancing passage. I think the phrase "phenotypically stable" to describe the overall history of the cell line in the Introduction may be too strong though and should be avoided.

*Answer: The sentences containing the term "phenotypically stable" have been reworded (abstract and page 13, first paragraph).*

It may be more accurate to mention a few studies, such as with calcification where they did share phenotype with primary cells. In addition, this cell line was immortalized with viral methodology which altered other phenotype changes such as loss of senescence.

*Answer: Some more recent studies have been added in the part of the introduction dealing with MOVAS cells as well as in the discussion section (see references 13, 15, 16, 17, 21, 22, 29, 30 and 41).*

**Reviewer #3:**

Dear authors,

thank you for your submission. Your study adds important knowledge to this field and is of special interest to research performing basic studies on MOVAS.

*We thank Reviewer #3 for this nice comment.*

However, with the limited information you provide in your methods section most experiments seem difficult to reproduce (e.g., starting with the frequency of passaging of your cells). In addition a few things are missing to make your results and conclusions more reliable and scientifically sound.

*Answer: MOVAS cells were seeded at a density of 10^4^ cells/cm² and reached confluence in 3 or 4 days, which corresponded to a passing frequency of every 4 days. This information has been added in the revised manuscript (see paragraph 2.1.)*

For your statistics:

- power calculation (with the significance you show I have no doubts that your n-number is in an adequate range, yet a power calculation should be performed prior to your experiments)

*Answer: Initially, we were unable to perform a statistical power calculation because we did not have any preliminary data or data from the literature studying differences in contractile marker expression depending on culture passages in MOVAS cells. We therefore conducted the first experiments with a usual number of replicates (n=4) and then performed a power calculation using G-Power software to refine the number of replicates required.*

- correlation analysis between the contractile and synthetic gene expression levels > proof of a reverse correlation may support the hypothesis of a switch from contractile to synthetic over time

*Answer: As suggested by Reviewer #3, the evolution of the relative expression of the different markers as a function of the number of passages was compiled in a single graph (see below), which was added to the revised manuscript (Fig. 2, panel C).*

For your methods/results:

- staining protocol for figure 1 A is missing and selection of images might not be ideal

*Answer: The staining protocol was the same for all data presented in Figure 1. This protocol was completed in the Mat&Meth section. In addition, new figures of the cells at P3, P5 and P8 were proposed.*

- stability of your reference gene expression over the different passages must be tested

*Answer: The stability of reference gene expression has been tested and no variation of EEF1A1 expression between different passages was observed (see figure below). This information has been added in the manuscript (see paragraph 3.2.).*

- product length of PCR products missing in suppl. table, so does the inclusion of any internal PCR controls

*Answer: Length of PCR products has been added in the table S1 (supplemental data file)*

For your discussion:

- ideally find studies that used MOVAS to study atherosclerosis specifically

*Answer: Some more recent studies have been added in the part of the introduction dealing with MOVAS cells as well as in the discussion section (see references 13, 15, 16, 17, 21, 22, 29, 30 and 41).*

Please also have a look at the attached word document that provides more detailed explanations in the comments.

**Responses to the comments addressed by Reviewer #3 in the word document:**

Page 2: Introduce acronym (AFM) here?

*Answer: We should limit the use of acronyms in the abstract (especially since the term is only mentioned once), which is why this abbreviation was introduced in the corresponding Mat&Meth section (the first time AFM appears in the article).*

Page 3: You have used Acta2, which to my knowledge is not only specific to the aorta but to smooth muscle cells in general.

*Answer: Reviewer #3 is right, we have followed his/her recommendation and removed the adjective ‘aortic’.*

Page 3: Upon alteration of their microenvironment indeed they seem to take on characteristics specific to other vascular cell types. Even though it has been hypothesized previously that specific VSMC have an innate stem cell-like ability to differentiate it still has not been shown whether they change their SMC identity holistically.

*Answer: We agree with Reviewer #3 to qualify this statement and use the term "subtypes", as suggested, which seems more appropriate.*

Page 3: Consider 1-2 additional references in this section to highlight the incidence of atherosclerosis and that it often also occurs due to aging.

*Answer: This paragraph has been completed by mentioning the increasing incidence of atherosclerosis and cardiovascular diseases, as well as the role of ageing as a contributing factor for its development.*

Page 3: Is this section relevant for the study?

*Answer: This section may be not relevant to this study, but we feel it is important to include it for readers who are unfamiliar with the atherosclerotic process, as it is an essential and decisive step of its initiation.*

Page 4: Are there more recent studies using this cell line? Consider a paper studying atherosclerosis using MOVAS

*Answer: More recent studies have been cited (ref. 12 to 17).*

Page 5: So that technically means they different vials could have been in culture for different periods in advance ?

*Answer: In principle, the answer is yes. We do not control the number of passages undergone by the cells before commercialization by ATCC. In order to limit this variability, we used several different cell vials from the same batch.*

*This is why we took care in the conclusion not to give recommendations on the maximum number of passages for using these cells, but rather a general message to readers warning them that it is necessary to (i) characterize the phenotype of the cells at the time of thawing and (ii) monitor the evolution of this phenotype with increasing number of passages in order to avoid using cells that have changed phenotypically for in vitro experiments.*

*A paragraph describing the limitations of the study and specifically addressing this problem has been added at the end of the discussion section of the revised manuscript.*

Page 5: Passaging frequency in days? Consistent for all experiments? Where cells seeded at the same density?

*Answer: The passaging frequency of cells was 4 days, was consistent for all experiments and did not vary between the different cell vials tested. The cells were always seeded at the same density (10^4^ cells per cm²). The information about passaging frequency has been added in the revised manuscript (see paragraph 2.1.).*

Page 5: Did you also measure a -RT control and a water control?

*Answer: Yes, one well without RT product and another one containing only water were systematically used as negative controls for each RT-qPCR experiments.*

Page 5: Did you check for the stability of the housekeeping gene expression over the different passages? An important requirement of reference genes is them being unaffected by the experimental setup.

*Answer: Please see the response above about the stability of reference gene expression.*

Page 5: You used the SYBR green approach. How did you check the correctness of your products? Did you sequence them? Did you check for your product length? Add in your suppl. File the product length. Ideally this should be in a comparable range across all genes you compare with one another

*Answer: The PCR products were not sequenced, but their size ranged from 70 to 200 bp, in accordance with the recommendations for the use of SYBR Green technology. The primers were designed and verified in silico, then their efficiency was tested using several ranges of cDNA. For each PCR well, the specificity of the product was checked by analyzing the melting curve, and in each case, a single expected peak was observed, without dimer formation.*

*PCR product lengths were added in Table S1.*

Page 6: What was the concentration of the probe & DAPI and for how long did you incubate the cells with either?

*Answer: More information about actin probe and DAPI has been added in the Mat&Meth section (see the corresponding response above)*

Page 6: Of all cells captured? Randomly picked cells?

*Answer: The reported actin fluorescence represents the average fluorescence intensity for all cells of the field from randomly taken images.*

Page 6: Acronym has not been introduced thus far. Consider introducing it in methods section page 2.

*Answer: See our comment above about AFM abbreviation.*

Page 6: Did you coat these glass vials with something to facilitate the cells’ attachment to the surface?

*Answer: Coating the glass cups was not necessary for the AFM studies. The cells adhered well to this surface.*

Page 6: From my perspective it is not sufficiently explained how YM values were calculated. Especially relevant for an audience that is not familiar with this technique.

*Answer: Further details about AFM calibration and the calculation of YM values can be found in the supplemental data file.*

Page 7: Concentration?

*Answer: Concentration of crystal violet (0.1% (m/v)) has been added.*

Page 7: Of each passage?

*Answer: Yes. The sentence has been changed as follows: "* *At days 1, 3, and 6, MOVAS cells of each passage were fixed using a 1.1% (v/v) glutaraldehyde solution prepared in DPBS at room temperature (20°C) for 20 min."*

Page 8: Did you perform a power calculation in advance to check if your n-number is high enough to proof your hypothesis? If so, please add here. In case you always show SD and not SEM for your errors you could consider mentioning this here. In that case you don’t need to mention it in each figure.

*Answer: Please see our response above about power calculation. Furthermore, we have always represented the data as means +/- SD. This information has been added to the paragraph corresponding to the statistical analysis in Mat&Meth section and has been removed from each figure legend.*

Page 8: Regarding the shape and overall appearance I agree. The actin staining however does not seem as convincing. It appears quite even throughout the cytosol and slightly oversaturated and therefore the typical fibrous staining pattern of actin is least visible in P3. Maybe you find better examples here.

*Answer: We have tried to select more representative images for the revised version of the manuscript.*

Page 9: Is the order of this figure correct? Because appearance wise (at least according to figure 1 A) P8 seems to show the biggest area covered by the cell body ?

*Answer: We have tried to select more representative images for the revised version of the manuscript.*

Page 9: Are the staining protocols for A and B the same? I mention this because in figure 1 A actin staining seems most intense in P8. I am slightly confused because in the methods section you mention Alexa Red as the probe and you suddenly show green images?

*Answer: Yes, it is the same protocol for all pictures used for figure 1 and all stainings were made within the same day. We have tried to select more representative images for the revised version of the manuscript.*

*This is indeed an actin-red probe, which emits red light (excitation: 540 nm; emission: 565 nm). The images were digitally reprocessed to convert the red pixels to green to make the images more visually appealing. Red labelling is often less noticeable to the naked eye than green. This information has been added in paragraph 2.3.*

Page 9: n-numbers missing! Why is the n-number of the actin labelling so much higher than the other parameters?

*Answer: n numbers have been added to each figure legend. Regarding actin labelling, we also had eight wells, as for the other parameters, but we took two pictures per well. For each picture, we measured the average fluorescence intensity for all the cells in the field. Therefore, in total, we therefore had 16 measurements per condition. To be more consistent in presenting our results, we averaged the values per well to present 8 values per condition. This new histogram is now presented in the revised version of Figure 1.*

Page 9: Stick with the same spelling as before, in introduction Krueppel.

*Answer: The correct spelling "Krüppel-like factor 4" has been now used throughout the text.*

Page 9: You should in your case definitely assess whether your reference gene expression remains stable over these passages.

*Answer: Please see our response above.*

Page 10: Consider a correlation test between the contractile and synthetic genes you tested. They seem to be reverse correlated.

*Answer: Please see our response above (a new panel has been added to figure 2).*

Page 10: n-number?

*Answer: n-number has added to the figure legend.*

Page 10: Consider highlighting this with arrows in the Figure.

*Answer: Arrows showing cytoskeleton actin fibers have been added in figure 3A.*

Page 10: Please add the equation how the YM values were calculated in the methods section. And in addition to that briefly describe what a low/high YM value indicates.

*Answer: Further details regarding AFM calibration and the calculation of YM values can be found in the supplemental data file. Besides, the significance of YM values in relation to cell stiffness had already been mentioned in paragraph 3.3.*

Page 10: Figure legend for 3 B: Number of experiments and statistical analysis you performed is missing. So do the p-values and whether SD or SEM was shown.

*Answer: n number has been added as well as the significance of the p-value (**:p<0.01) in the figure legend. Details about the presentation of values (mean ± SD) and the statistical tests have been added to paragraph 2.8.*

Page 11: Did you notice an increase in the time it takes to detach the cells for passaging?

*Answer: No, we did not observe any differences when detaching the cells for passaging.*

Page 11: In the methods section you refer to it as gap closure. I think for consistency you should stick with this term. Does this assay really allow to assess wound healing or just migration. Would healing would require a different type of assay, for instance a scratch assay.

*Answer: We agree with Reviewer #3, this is not really a scratch assay or a wound healing assay. The term "gap closure" seems more appropriate and has now been used throughout the text.*

Page 12: n-numbers

*Answer: n-numbers have been added in the figure legend.*

Page 12: At the level of expression they even seem to change upon isolation from the vessel of origin. Detachment from neighbouring cells seems to be powerful trigger for phenotype switching.

*Answer: Reviewer #3 is right. It seems important to explain this point further. We have incorporated his/her comment and added one reference (ref 26) to complete this part of the discussion.*

Page 14: No significant difference between P3 and P5, in fact it almost seems as if the adhesion in P5 would go down > do you have an explanation why this is?

*Answer: No, we have no explanation for this decrease, which remains not statistically significant.*

Page 14: Did you try staining your cells for proliferation markers e.g., Ki67

*Answer: No, we did not carry out any additional tests, such as Ki67 labelling, to assess proliferation.*

Page 15: Come to a conclusion in terms of your own experiments first. Ask yourself the question whether or not you would be confident that P3 would yield reliable results to study contractility or whether it would be worth using always the same passage. Maybe add a small outlook how you would for instance compare these cells to their counterparts in tissue?

*Answer: Conclusion has been reworded in order to take into account the comments of Reviewer #3.*
