# Supplementary material for: Evidence for loss of contractile phenotype of the mouse aortic vascular smooth muscle (MOVAS) cell line with increasing number of passages in vitro
Source: PLoS One. 2025 Dec 19;20(12):e0339118. doi: 10.1371/journal.pone.0339118 (PMC12716690; doi:10.1371/journal.pone.0339118)
Supplement: Attachment — Submitted filename: Response to reviewers-PONE-D-25-33029R1.docx [file pone.0339118.s007.docx]

**PONE-D-25-33029R1**

Cadoret et al. - *Evidence for loss of contractile phenotype of the mouse aortic vascular smooth muscle (MOVAS) cell line with increasing number of passages in vitro*.

Responses to reviewers:

**Reviewer #3:**

Results:

> PCR results:

Addition to Figure 2C: I appreciate that you decided to show the progression of your genes of interest over the different passages.

However, proof of linear correlation requires a statistical test (to my knowledge the standard test is the Pearson Correlation test that allows you to calculate an R-value). I suggest you add the calculated R value and the correlation graph to the supplements and to comment on the correlation in the figure legend of 2C.

*Answer: As suggested by Reviewer #3, regression lines and Pearson correlation coefficients were calculated and presented in supplemental data file (Fig. S1). R-values were also mentioned in the main text (page 10).*

*Here is Fig. S1:*
