# Supplementary material for: Evidence for loss of contractile phenotype of the mouse aortic vascular smooth muscle (MOVAS) cell line with increasing number of passages in vitro
Source: PLoS One. 2025 Dec 19;20(12):e0339118. doi: 10.1371/journal.pone.0339118 (PMC12716690; doi:10.1371/journal.pone.0339118)
Supplement: Attachment — Submitted filename: Response to reviewers-PONE-D-25-33029R3.docx [file pone.0339118.s008.docx]

**PONE-D-25-33029R3**

Cadoret et al. - *Evidence for loss of contractile phenotype of the mouse aortic vascular smooth muscle (MOVAS) cell line with increasing number of passages in vitro*.

Responses to reviewers:

**Reviewer #3:**

Results:

Dear authors,

Thank you for resubmitting your manuscript. I apologize for my probably vague instructions regarding the correlation analysis last time but it requires adaptation. I would suggest you to consider seeking support from a biostatistician.

What you need to correlate is the relative expression levels of the genes that indicate a contractile phenotype with the relative gene expression levels of the genes that indicate a more synthetic phenotype. You basically do this to support the conclusion that the expression of contractile genes decreases whilst at the same time the expression of synthetic genes increases and to answer the question if their expression is somewhat connected/correlated with one another.

You may require a specific correlation test to do that, as your expression levels might be inversely correlated.

If you can show that it would be a suppotive and strong statistical evidence for your conclusions.

I hope this was helpful.

Best regards.

The reviewer.

*Answer: We thank the Reviewer 3 for these clarifications. We believe we have met his/her expectations by establishing correlations between contractile and synthetic markers. This representation is indeed interesting and very useful, as it clearly shows that the expression of contractile markers decreases concomitantly with the increase in the expression of synthetic markers. We applied a Pearson test, which shows that these correlations are statistically significant (p-values and correlation coefficients (r) are shown in the corresponding figure).*

*This new figure has been added in supplemental data file and has also been mentioned in the text.*

*Here is Fig. S1:*

*Fig. S1. Relative expression of contractile vs synthetic markers in MOVAS cells. RT-qPCR analysis of gene expression of contractile (Acta2, Cnn1, Myocd) vs synthetic (Lgals3, Klf4) phenotype markers in MOVAS cells at different passages were pooled in the same plot. Regression lines were calculated and a Pearson correlation test was used to determine the statistical significance and the r coefficient (indicated within each graph). The regression lines are represented by black lines, with 95% confidence intervals shown in grey.*
